# Assessment of air quality using a cloud model method

**DOI:** 10.1098/rsos.171580

**Published:** 2018-09-26

**Authors:** Qingwei Xu, Kaili Xu

**Affiliations:** School of Resources and Civil Engineering, Northeastern University, Shenyang 110819, People's Republic of China

**Keywords:** air quality assessment, cloud model, air pollution indicator, cloud generator, seasonal variation

## Abstract

To effectively control air pollution, it is necessary to obtain a preliminary assessment of air quality. The purpose of this study was to introduce a cloud model method in air pollution assessment. First, the standard cloud models of air pollution indicators were obtained, and the calculating process of numerical characteristics employed by the standard cloud model was explained. Second, the levels of air pollution indicators were presented based on the qualitative and quantitative analysis of cloud models, which realized the uncertainty conversion between qualitative concepts and their corresponding quantitative values, as well as taking the fuzziness and randomness into account. Air quality assessment results including SO_2_, NO_2_, CO, O_3_, PM_10_ and PM_2.5_ were analysed. Third, the cloud model adopted in the assessment process of air quality was validated by grey relational analysis, and the results confirmed the validity of cloud model assessment. Fourth, the air pollution level of the air quality index (AQI) was determined, and the fuzziness and randomness of the assessment results were thoroughly analysed by taking entropy and hyper entropy into consideration. Fifth, seasonal variations in different air pollution indicators were analysed to proffer a series of recommendations for government policy decision-makers and travellers. The cloud model provided a new method for air quality assessment.

## 1. Introduction

China's economy has rapidly expanded since the Reform and Opening policy [1] and is now the world's second largest. The improved living standards accompanied by this economic growth have, unfortunately, also resulted in severe environmental effects [2,3], which are dangerous to both the economy and human health [4]. To effectively control air pollution, it is first necessary to thoroughly and scientifically assess the current air quality [5,6].

Heavy element pollution can be divided into natural and anthropogenic pollution sources [7]. Not only airborne heavy elements but also gaseous, semivolatile and particulate matter have both natural and anthropogenic pollution sources. Natural air pollution sources include volcanic eruptions [8] and forest fires [9]. Anthropogenic air pollution sources may be fixed or moving: fixed pollution sources mainly include chimneys [10] and power generation stations [11], while moving pollution sources mainly include automobiles [12], trains [13] and steamships [14].

There are many methods for air quality assessment, mainly including dynamic models [15–17], fuzzy assessments [18–20], integrated assessment methodologies [21,22], principal component analysis [23–25] and grey system theory [26,27]. Thunis & Clappier [16] proposed common indicators and diagrams which have since proven useful for determining the magnitude of locally produced emissions effects. Carbajal-Hernandez *et al*. [19] adopted a fuzzy inference system to perform parameter classifications, which they integrated into an air quality index (AQI) that describes pollution levels. Miranda *et al*. [22] developed an integrated assessment model of the cost-effectiveness and health effects of emissions reduction practices. Through data reduction, principal component analysis reveals recurring and independent modes of variations within a very large dataset, thereby summarizing the essential information of that dataset to yield meaningful and descriptive conclusions [23]. A grey relational model was also successfully applied to air quality assessment in high-traffic areas in Shanghai, China [27].

Despite the wealth of research on air quality assessment, there are many complex factors that cause air pollution and the existing air quality assessment methods are generally imprecise and plagued by randomness [28]. Yadav *et al*. [20] proposed a fuzzy-genetic algorithm approach to deal with the randomness and fuzziness in air quality assessment. They also investigated the efficacy of fuzzy set theoretic method in combination with genetic algorithm in describing air quality in linguistic terms with a linguistic degree of certainty attached to each description via Zadeh-Deshpande approach. The uncertainty indicates error in data with linguistic term transformation and randomness indicates uncertainty degree that can be accepted in data. Yang & Fan [27] used a grey relational model to manage uncertainty in air quality assessment. Fuzzy genetic algorithm and grey relational methods, however, do not resolve the uncertainty, randomness and fuzziness in given air quality assessment at the same time.

There is urgent demand for new methods of assessing air quality which take uncertainty, randomness and fuzziness fully into consideration. The cloud model proposed by Li and co-workers [29,30] represents a new approach to air quality assessment wherein the uncertainty between qualitative concepts and quantitative values is converted while the fuzziness and randomness are taken into account simultaneously. Cloud models have been applied to the assessment in petrochemical enterprise [31], railway container station selection [32], water allocation [33] and stability analysis [34]. In this study, a cloud model was used to assess air pollution.

The purpose of this study was to establish a cloud model for air pollution assessment. The detailed calculation processes of cloud model, and relevant air pollution levels were discussed below. Grey relational analysis was applied to the air pollution indicator assessment to validate the introduced cloud model. The seasonal variation characteristics of different air pollution indicators were also analysed in depth.

## 2. Methods

### 2.1. Cloud model concept

The cloud model is an uncertainty transformation between a qualitative concept and its corresponding quantitative representation. Uncertainty transformations are associated with fuzziness and randomness.

Given a qualitative concept *C* defined over a universe domain of discourse *U*, let *x* ∈ *U* be a random element of concept *C* and *μ*(*x*) ∈ [0,1], the membership of *x* belonging to *C*, which corresponds to a random number with a steady tendency, that is

2.1 μ: U→[0, 1]∀x∈U, x→μ(x).

The distribution of *x* in the universe *U* can then be defined as ‘cloud’ and *x* as the ‘cloud drop’. A random number exists in the sense of probability theory, whereas the membership lies in the sense of fuzzy set theory. Thus, ∀*x* ∈ *U* and the mapping *μ*(*x*) is a one-to-many mapping in nature; in other words, the membership of *x* belonging to the concept *C* is a probability distribution rather than a fixed number. This allows the cloud model to effectively integrate the randomness and fuzziness of concepts.

**Figure 1.**
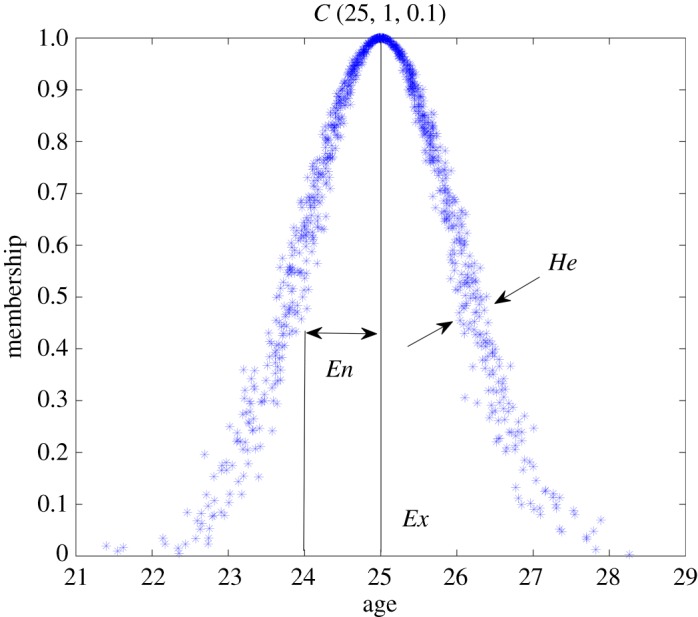
Cloud model of *Youth* with 1000 cloud drops.

The cloud model describes the overall quantitative features of a concept by three numerical characteristics (*Ex, En, He*). The cloud model of *Youth* is used below as an example to explain the meaning of each numerical characteristic (figure 1).

Expectation *Ex* is the mathematical expectation of the cloud model belonging to a concept in the universal system, and *Ex* is also the most representative cloud drop for the qualitative concept, which is the centre value of the qualitative concept. Twenty-five years old is most representative of the concept of ‘*Youth*’ in figure 1.

Entropy *En* is the uncertainty distribution of the concept representing the range of values that could be accepted in the universe, which reflects the fuzziness of the qualitative concept and is used to measure the randomness of cloud drops.

Hyper entropy *He* is the uncertainty degree of entropy *En*, reflects the dispersion of cloud drops and determines the cloud thickness. A larger *He* indicates greater randomness of the membership degree and cloud thickness.

### 2.2. Forward cloud generator

The cloud generator establishes a mapping relationship between the qualitative concept and its corresponding quantitative characteristic. The most important algorithms of the cloud model are forward and backward cloud generators. The forward cloud generator maps the qualitative concept to its corresponding quantitative characteristic, producing as many cloud drops as needed when the three numerical characteristics (*Ex*, *En*, *He*) are provided. It can be easily qualitatively analysed by mapping the cloud model and standard cloud models into one picture. The algorithm of the forward cloud generator is as follows.

Input: the numerical characteristics (*Ex*, *En*, *He*) of the qualitative concept and the number of cloud drops *n*.

Output: the membership degree, *μ*(*x*).

(1) Generate a normally distributed random number *En′* with mean *En* and standard deviation *He*.
(2) Generate a normally distributed random number *x* with mean *Ex* and standard deviation *En*.
(3) Let *x* be a specific quantitative value of the qualitative concept.
(4) Calculate $\mu (x)={\text{e}}^{-{\left(x-Ex\right)}^{2}/2{\left(En^{\prime }\right)}^{2}}$.
(5) Repeat steps 1–4 until *n* cloud drops are generated.

### 2.3. Backward cloud generator

Unlike the forward cloud generator, a backward cloud generator maps the quantitative characteristic to qualitative concept, and determines the three numerical characteristics (*Ex, En, He*) to represent the corresponding qualitative concept from given cloud drops. From the given cloud drops, the numerical characteristics (*Ex*, *En*, *He*) can be obtained according to the backward cloud generator. The algorithm of the backward cloud generator is as follows.

Input: cloud drops *x_i_* (*i* = 1, 2, … , *n*);

Output: numerical characteristics (*Ex*, *En*, *He*) of cloud drops.

(1) $Ex=\frac{1}{n}\sum_{i=1}^{n}x_{i}$,
(2) $En=\sqrt{\frac{\pi }{2}}\times \frac{1}{n}\sum_{i=1}^{n}\left|x_{i}-Ex\right|$,
(3) $He=\sqrt{\left|\frac{1}{n-1}\sum_{i=1}^{n}{\left(x_{i}-Ex\right)}^{2}-En^{2}\right|}$.

### 2.4. Standard cloud model

It is necessary to transform the air pollution level into a standard cloud model to assess air quality. For an indicator with bilateral constraints, the numerical characteristics of the standard cloud model can be calculated as follows:

2.2 $$Ex=\frac{C_{\max}+C_{\min}}{2},$$

2.3 $$En=\frac{C_{\max}-C_{\min}}{6}$$

2.4 andHe=k×En,

where *C*_max_ and *C*_min_ are the maximum and minimum of the concentration range of an air pollution indicator, respectively, and *k* a constant that changes according to the randomness and fuzziness of different indicators [35]. A larger *He*, as mentioned above, indicates greater randomness of assessment indicators; a smaller *He* suggests less randomness of assessment indicators and randomness that is more easily lost [36]. Usually, *k* is no more than one-third; *k* = 0.1 in this treatment.

### 2.5. Similarity between cloud model and standard cloud model

When the cloud model of an air pollution indicator assessment is achieved according to the given cloud drops, it is necessary to calculate the similarity between the specific cloud model and a standard cloud model to determine to which air pollution level the specific cloud model belongs. The similarity between the specific cloud model and a standard cloud model is calculated as follows:

2.5 $$\lambda_{j}={\text{e}}^{-{\left(Ex-Ex_{j}\right)}^{2}/2{\left(En_{j}\right)}^{2}},$$

where *Ex* is the specific cloud model expectation of the air pollution indicator, *Ex_j_* is the expectation of the *j*th standard cloud model and *En_j_* is the entropy of the *j*th standard cloud model.

When the similarity *λ_j_* between the specific and standard cloud models is calculated on the basis of equation (2.5), the standard cloud model corresponding to the maximum similarity *λ_j_* is the quantitative assessment result based on the air pollution indicators assessed and the maximum membership degree principle.

### 2.6. AQI calculation method

The individual AQI (IAQI) of the air pollution indicator *P* is calculated as follows:

2.6 $$\text{IAQ}{\text{I}}_{P}=\frac{\text{IAQ}{\text{I}}_{Hi}-\text{IAQ}{\text{I}}_{Lo}}{\text{B}{\text{P}}_{Hi}-BP_{Lo}}(C_{P}-\text{B}{\text{P}}_{Lo})+\text{IAQ}{\text{I}}_{Lo},$$

where IAQI*_P_* is the IAQI of air pollution indicator *P*, *C_P_* is the concentration of *P*, BP*_Hi_* is the concentration breakpoint which is higher than *C_P_*, BP*_Lo_* is the concentration breakpoint which is lower than *C_p_*, IAQI*_Hi_* is the IAQI breakpoint corresponding to BP*_Hi_*, and IAQI*_Lo_* is the IAQI breakpoint corresponding to BP*_Lo_*.

AQI can be calculated as follows:

2.7 $$\text{AQI}=\max\{\text{IAQ}{\text{I}}_{1},\;\text{IAQ}{\text{I}}_{2},\;\ldots ,\;\text{IAQ}{\text{I}}_{n}\},$$

where *n* is the number of air pollution indicators.

## 3. Results

### 3.1. Air pollution level

China has released new air quality standards to safeguard its citizens' living environment and resolve air pollution issues, including the Ambient Air Quality Standards (GB 3095-2012) and Technical Regulation on Ambient Air Quality Index (HJ 633-2012) [37]. Air pollution indicators such as SO_2_, NO_2_, CO, O_3_, PM_10_ and PM_2.5_ are generally used to assess air quality under these new standards. The concentration ranges and levels of different air pollution indicators are shown in table 1.

**Table 1.** Concentration ranges and level of air pollution indicators.

level	AQI	PM_2.5_ (μg m^−3^)	PM_10_ (μg m^−3^)	SO_2_ (μg m^−3^)	CO (μg m^−3^)	NO_2_ (μg m^−3^)	O_3_ (μg m^−3^)
1. Good	50	35	50	50	2	40	100
2. Moderate	100	75	150	150	4	80	160
3. Lightly Polluted	150	115	250	475	14	180	215
4. Moderately Polluted	200	150	350	800	24	280	265
5. Heavily Polluted	300	250	420	1600	36	565	800

Table 1 shows AQI values from 0 to 300. The concentration ranges and concentrations of air pollution indicators are specifically confined by the new air quality standards. It is necessary to calculate the AQI to determine the air quality for a given area, so the air pollution concentrations in table 1 were transformed to standard cloud models according to equations (2.2)–(2.4); the results are shown in table 2.

**Table 2.** Standard cloud model of air pollution indicators.

level	Good	Moderate	Lightly Polluted	Moderately Polluted	Heavily Polluted
AQI	(25, 8.33, 0.83)	(75, 8.33, 0.83)	(125, 8.33, 0.83)	(175, 8.33, 0.83)	(250, 16.67, 1.67)
PM_2.5_	(17.5, 5.83, 0.58)	(55, 6.67, 0.67)	(95, 6.67, 0.67)	(132.5, 5.83, 0.58)	(200, 16.67, 1.67)
PM_10_	(25, 8.33, 0.83)	(100, 16.67, 1.67)	(200, 16.67, 1.67)	(300, 16.67, 1.67)	(385, 11.67, 1.17)
SO_2_	(25, 8.33, 0.83)	(100, 16.67, 1.67)	(312.5, 54.17, 5.42)	(637.5, 54.17, 5.42)	(1200, 133.33, 13.33)
CO	(1, 0.33, 0.03)	(3, 0.33, 0.03)	(9, 1.67, 0.17)	(19, 1.67, 0.17)	(30, 2, 0.2)
NO_2_	(20, 6.67, 0.67)	(60, 6.67, 0.67)	(130, 16.67, 1.67)	(230, 16.67, 1.67)	(422.5, 47.5, 4.75)
O_3_	(50, 16.67, 1.67)	(130, 10, 1)	(187.5, 9.17, 0.92)	(240, 8.33, 0.83)	(532.5, 89.17, 8.92)

Air pollution indicators can be qualitatively assessed in the cloud model by means of a forward cloud generator, which maps the cloud model of an air pollution indicator and its corresponding standard cloud model into a cloud picture. The air pollution indicator can be qualitatively stated according to the position into which the cloud picture falls in the standard cloud model.

### 3.2. Air pollution indicator cloud model

Shenyang is the capital of Liaoning province, an important industrial base in China, and also the central city of Northeast China as approved by the State Council. Shenyang covers more than 12 948 km^2^ and had a resident population over 8.29 million in 2017. In this study, its air pollution indicators were assessed by the introduced cloud model using data from November 2016 (table 3).

**Table 3.** Air pollution data: Shenyang, November 2016.

date	PM_2.5_ (μg m^−3^)	PM_10_ (μg m^−3^)	SO_2_ (μg m^−3^)	CO (mg m^−3^)	NO_2_ (μg m^−3^)	O_3_ (μg m^−3^)
1 Nov	55.5	94.6	87.3	1.071	48.1	57
2 Nov	48.2	85.3	90.3	1.229	57.8	64
3 Nov	64.2	107.8	102.7	1.138	63	32
4 Nov	120.9	184.9	125	1.554	63.2	47
5 Nov	220.2	274.4	47.1	1.188	54.7	100
6 Nov	95.8	116.2	53	0.683	47.3	74
7 Nov	169.4	191.5	48.9	0.946	49.9	89
8 Nov	60.3	74.3	55.3	0.596	46.2	60
9 Nov	66.7	99	98.6	1.146	58.9	44
10 Nov	31.5	52	52.8	0.754	35.8	56
11 Nov	48.1	67.9	51.3	0.679	41.7	47
12 Nov	50.2	80.9	63.8	0.809	47.9	57
13 Nov	64.2	105.4	83.9	1.154	47.8	68
14 Nov	48.7	109.9	34.6	0.625	28.1	69
15 Nov	33.6	62.4	47.6	0.578	39.4	56
16 Nov	86.7	134.7	104.3	1.47	58.4	40
17 Nov	179.5	247.8	108.5	1.975	71.9	62
18 Nov	82.4	125.7	55	0.957	41.4	70
19 Nov	36.8	47.9	32.2	0.563	28.4	50
20 Nov	33.6	44.2	54.6	0.596	39.5	55
21 Nov	19.7	32.6	40	0.479	27.5	56
22 Nov	20.8	36	40.6	0.488	29	60
23 Nov	51	81	82	0.908	49.8	48
24 Nov	74	111.8	88.8	1.267	54.2	62
25 Nov	66.5	108.9	107.4	1.546	56	48
26 Nov	106.5	157.1	70.3	1.367	55.8	38
27 Nov	77.5	119.7	59	1.221	47.2	70
28 Nov	65.6	104.4	89.9	1.246	59.3	47
29 Nov	68.7	106.7	106.5	1.033	59.3	58
30 Nov	58.3	88.4	58.5	1.125	46.4	53

The cloud model of air pollution indicators was established with a backward cloud generator in Matlab software. The Matlab code of forward and backward cloud generators is available in the literature [31]. The air pollution data in table 3 were transformed into cloud models, as shown in table 4.

**Table 4.** Cloud model and air pollution indicators (levels as described in table 1).

month	PM_2.5_		PM_10_		SO_2_	
	cloud model	level	cloud model	level	cloud model	level
Nov 2016	(73.50, 39.93, 23.36)	2	(107.45, 48.57, 29.08)	2	(71.36, 29.07, 12.65)	2
Dec 2016	(95.65, 53.36, 10.47)	3	(133.52, 63.70, 7.35)	2	(95.65, 39.70, 7.42)	2
Jan 2017	(88.84, 56.46, 9.74)	3	(128.03, 64.97, 12.23)	2	(98.77, 41.92, 16.19)	2
Feb 2017	(66.18, 37.95, 6.10)	2	(95.75, 42.97, 3.16)	2	(70.96, 31.64, 5.87)	2
Mar 2017	(83.03, 45.81, 16.47)	3	(124.23, 55.18, 17.52)	2	(54.10, 23.10, 8.11)	2
Apr 2017	(47.87, 24.53, 8.89)	2	(104.13, 34.35, 10.86)	2	(23.93, 11.27, 3.23)	1
May 2017	(40.94, 15.04, 4.08)	2	(107.03, 60.05, 32.71)	2	(14.13, 4.86, 1.78)	1
Jun 2017	(34.33, 14.12, 3.46)	1	(64.00, 24.06, 6.25)	2	(18.30, 6.37, 0.93)	1
Jul 2017	(30.94, 10.37, 2.33)	1	(51.97, 15.16, 0.93)	2	(12.23, 5.40, 0.78)	1
Aug 2017	(25.03, 9.10, 6.32)	1	(46.00, 14.64, 7.64)	1	(14.97, 4.49, 2.03)	1
Sep 2017	(31.97, 12.32, 1.96)	1	(62.20, 16.24, 2.60)	2	(22.57, 7.02, 1.78)	1
Oct 2017	(51.94, 33.77, 1.28)	2	(88.90, 44.89, 6.90)	2	(24.74, 12.46, 3.97)	1

	CO		NO_2_		O_3_	
month	cloud model	level	cloud model	level	cloud model	level
Nov 2016	(1.01, 0.38, 0.08)	1	(48.46, 11.17, 2.21)	2	(57.90, 12.81, 6.10)	1
Dec 2016	(1.44, 0.65, 0.22)	1	(57.42, 16.10, 6.21)	2	(39.77, 14.41, 2.56)	1
Jan 2017	(1.39, 0.69, 0.26)	1	(54.81, 20.87, 8.33)	2	(50, 16.17, 4.49)	1
Feb 2017	(1.07, 0.52, 0.02)	1	(46.14, 16.38, 6.26)	2	(75.68, 12.64, 7.94)	1
Mar 2017	(1.02, 0.42, 0.12)	1	(53.71, 17.76, 6.26)	2	(109.35, 18.60, 6.92)	2
Apr 2017	(0.77, 0.28, 0.07)	1	(36.37, 9.23, 0.97)	1	(119.63, 29.06, 5.34)	2
May 2017	(0.63, 0.20, 0.02)	1	(29.13, 8.11, 0.62)	1	(144.16, 47.51, 9.38)	2
Jun 2017	(0.77, 0.30, 0.05)	1	(33.17, 8.06, 1.35)	1	(156.13, 52.78, 13.02)	2
Jul 2017	(0.74, 0.19, 0.07)	1	(27.58, 10.45, 2.11)	1	(155.23, 38.49, 12.38)	2
Aug 2017	(0.83, 0.26, 0.10)	1	(29.74, 6.38, 3.59)	1	(106.87, 38.48, 12.15)	2
Sep 2017	(0.93, 0.29, 0.08)	1	(37.93, 7.51, 0.82)	1	(102.93, 32.17, 11.98)	2
Oct 2017	(1.10, 0.60, 0.13)	1	(47.74, 18.87, 4.93)	2	(72.94, 23.59, 8.55)	1

**Figure 2.**
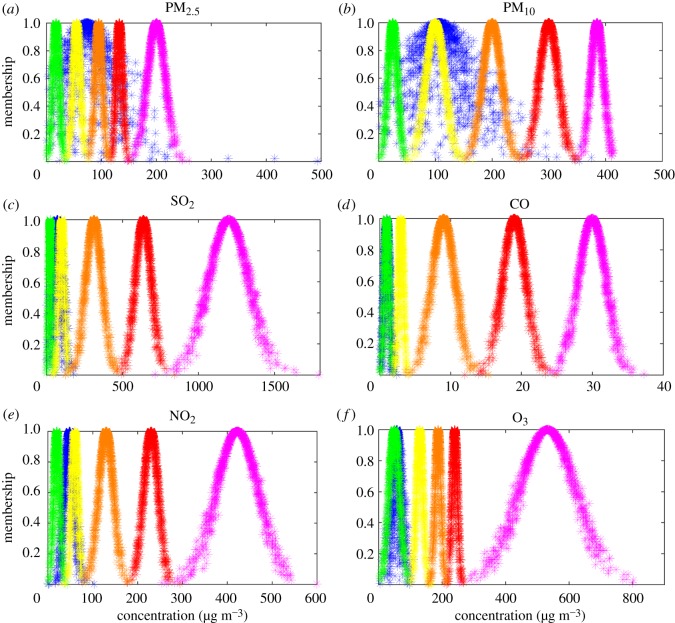
Air pollution indicator cloud models and corresponding standard cloud models. Green, yellow, orange, red and pink standard cloud models indicate Good, Moderate, Lightly Polluted, Moderately Polluted and Heavily Polluted levels, respectively. All air pollution indicator cloud models marked in blue.

A qualitative assessment was achieved by mapping of the cloud model for an air pollution indicator and its corresponding standard cloud models into a cloud picture in Matlab, as shown in figure 2.

If the expectation of the cloud model was relatively small but the entropy was relatively large, some cloud drops showed negative expectation (air pollution concentration) after being generated by the numerical characteristics due to the fuzziness of the cloud model. To ensure a practical cloud model assessment, the appropriate drawing area was selected according to the physical significance of air pollution indicators when drawing the cloud model images [38]. All air pollution indicators should be positive concentrations, therefore, all the drawing areas should also have positive values.

## 4. Discussion

### 4.1. Air pollution indicator assessment results

Consider the air pollution indicator of PM_2.5_ as an example. Its quantitative numerical characteristics are (73.50, 39.93, 23.36) and its qualitative concept is shown in figure 2*a*; it falls between Moderately and Lightly Polluted in the standard cloud models. That is to say, the qualitative assessment result of the air pollution indicator of PM_2.5_ lies between Moderate and Lightly Polluted.

The similarity between the cloud model of PM_2.5_ and its corresponding standard cloud models was calculated to determine the specific level to which it belonged via equation (2.5), with *λ*_2_ = 0.0216, *λ*_3_ = 0.0033 and others 0. The air pollution indicator of PM_2.5_ belonged to Moderate in the standard cloud model according to the maximum membership degree principle. That is to say, the quantitative assessment result of air pollution indicator of PM_2.5_ was Moderate. The qualitative assessment result of PM_2.5_ was between Moderate and Lightly Polluted, and the quantitative assessment result of PM_2.5_ was Moderate. Therefore, combining qualitative and quantitative assessment results, the air pollution level of PM_2.5_ was Moderate in Shenyang in November 2016.

The assessment results of air pollution indicators of PM_10_, SO_2_ and NO_2_ were also Moderate and CO and O_3_ were Good in Shenyang in November 2016. The main air pollution indicators in Shenyang during that month were PM_2.5_, PM_10_, SO_2_ and NO_2_. There were many factors that affected air quality and thus many remediation measures that may be taken to improve air quality.

All the assessment results of air pollution indicators were the same by comparison against the expectations and corresponding air pollution levels (table 1), which was in line with Xu *et al.*'s intuitive understanding that the assessment result of cloud model was mainly based on the expectation of indicator assessed [31]. Air quality was worse when the air pollution indicator expectation was poor, which confirms that the introduced cloud model yields accurate air quality assessments. A qualitative assessment was obtained by comparing the cloud model of each air pollution indicator and its corresponding standard cloud model. The cloud drops of PM_2.5_, as discussed above (figure 2*a*), are a good example of this, most of the cloud drops fall between Moderate and Lightly Polluted in the standard cloud models, indicating that the qualitative assessment result of PM_2.5_ was between Moderate and Lightly Polluted in Shenyang in November 2016. Greater cloud model coverage area also indicates greater fuzziness in determining the corresponding air pollution level and a longer time period of serious pollution than the expectation; in other words, the air pollution data were scattered across a very wide range and had large changes in air pollution levels. Air pollution indicators with greater cloud thickness also showed greater randomness; that is to say, the same air pollution concentration may have different membership degrees. For example, the membership degrees of cloud drops belonging to Lightly Polluted was from 0.6 to 0.9 in the case of air pollution indicator of PM_2.5_ at concentration 100 µg m^−3^ (figure 2*a*).

### 4.2. Comparison with grey relational analysis

Grey relational analysis is an important aspect of grey system theory, and usually used to calculate the grey relational degree among different factors [39,40].

Let the actual monitoring value of an air pollution indicator be *A* = {*a_j_*} 1 < *j* < *n*, where *n* is the number of air pollution indicators. *a_j_* is the expectation of the actual monitoring value of the air pollution indicator. Let the limiting value of air pollution indicator be *B* = {*b_ij_*} 1 < *i* < *m*, where *m* is the number of air pollution levels. *b_ij_* is the expectation of the air pollution level in this paper.

In the assessment process, different dimensions of air pollution indicators may cause large differences in numerical values in the monitoring data. The air pollution indicators should be rendered dimensionless:

4.1 $$d_{j}=\frac{a_{j}}{b_{1j}}$$

and

4.2 $$f_{ij}=\frac{b_{ij}}{b_{1j}}.$$

After making the air pollution indicators dimensionless, the actual monitoring value of the air pollution indicator is transferred to *D* = {*d_j_*} and the limiting value of the air pollution indicator is transferred to *F* = {*f_ij_*}. By comparing *D* as the reference sequence and *F* as the sequence, the grey relational coefficient of the *j*th actual monitoring value of the *i*th limiting value can be calculated as follows:

4.3 $$\xi_{ij}=\frac{\underset{1\leq j\leq n}{\min_{1\leq i\leq m}}|d_{j}-f_{ij}|+\rho \underset{1\leq j\leq n}{\max_{1\leq i\leq m}}|d_{j}-f_{ij}|}{\left|d_{j}-f_{ij}|+\rho \underset{1\leq j\leq n}{\max_{1\leq i\leq m}}|d_{j}-f_{ij}\right|},$$

where *ρ* is resolution coefficient and usually set to 0.5 [39].

The grey relational degree of the air pollution indicator can be obtained as follows:

4.4 $$r_{j}=\max\{\xi_{ij}\}.$$

The corresponding level of parameter *r_j_* is the air pollution level of the *j*th air pollution indicator.

The main assessment process and results of air pollution indicators using grey relational analysis is shown below. The limiting values of air pollution indicators (table 1) were obtained as follows:

$$B=\left[\begin{array}{cccccc} 17.5 & 25 & 25 & 1 & 20 & 50\\ 55.5 & 100.5 & 100.5 & 3 & 60.5 & 180.5\\ 95.5 & 200.5 & 313 & 9 & 130.5 & 250.5\\ 133.5 & 300.5 & 638 & 19 & 230.5 & 350.5\\ 200.5 & 385.5 & 1200 & 30 & 423 & 600.5 \end{array}\right].$$

The column of matrix *B*, from left to right, indicates air pollution indicators of PM_2.5_, PM_10_, SO_2_, CO, NO_2_ and O_3_, respectively. The row of matrix *B*, from up to down, indicates air pollution levels of Good, Moderate, Lightly Polluted, Moderately Polluted and Heavily Polluted, respectively. The actual monitoring values of air pollution indicators (table 3) were gathered as follows:

A=[73.5108.4571.331.0148.4657.9].

A non-dimensionalized matrix of the limiting value of air pollution indicator follows according to equation (4.2):

$$F=\left[\begin{array}{cccccc} 1 & 1 & 1 & 1 & 1 & 1\\ 3.17 & 4.02 & 4.02 & 3 & 3.03 & 3.61\\ 5.46 & 8.02\; & 12.52 & \;9 & 6.53 & 5.01\\ 7.63 & 12.02 & 25.52 & 19 & 11.53 & 7.01\\ 11.46 & 15.42 & 48 & 30 & 21.15 & 12.01 \end{array}\right].$$

The actual monitoring value of each air pollution indicator was transferred to *D* = [4.2 4.34 2.85 1.01 2.42 1.16] based on equation (4.1).

The grey relational coefficients were calculated based on equation (4.3) as follows:

$$\xi =\left[\begin{array}{cccccc} 0.88 & 0.87 & 0.92 & 1 & 0.94 & 0.99\\ 0.96 & 0.99 & 0.95 & 0.92 & 0.97 & 0.9\\ 0.95 & 0.86 & 0.7 & 0.74 & 0.85 & 0.85\\ 0.87 & 0.75 & 0.5 & 0.56 & 0.71 & 0.79\\ 0.76 & 0.67 & 0.33 & 0.44 & 0.55 & 0.68 \end{array}\right].$$

The grey relational degrees of air pollution indicators were obtained based on equation (4.4) as follows:

$$r=[\xi_{21}\;\;\;\xi_{22}\;\;\;\xi_{23}\;\;\;\xi_{14}\;\;\;\xi_{25}\;\;\;\xi_{16}].$$

The air pollution levels of PM_2.5_, PM_10_, SO_2_, CO, NO_2_ and O_3_ were Moderate, Moderate, Moderate, Good, Moderate and Good, in Shenyang in November 2016, respectively. These results are consistent with the cloud model results, indicating that the cloud model indeed applies to the accurate assessment of air pollution indicators.

### 4.3. AQI assessment

**Figure 3.**
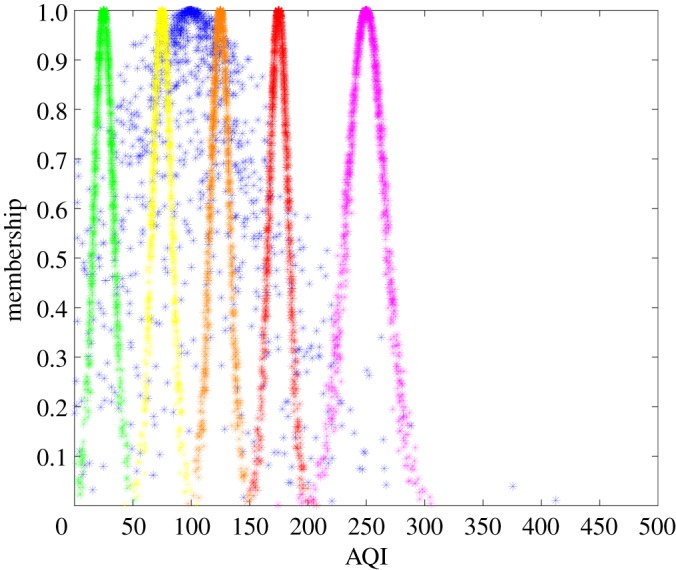
AQI cloud model and corresponding standard cloud models. Green, yellow, orange, red and pink standard cloud models indicate Good, Moderate, Lightly Polluted, Moderately Polluted and Heavily Polluted levels, respectively. Cloud model of AQI marked in blue.

The AQI is the maximum IAQI of air quality indicators and an important parameter for weather forecasting in China. The assessments of air quality indicators described above were used to calculate AQI. The IAQI of air quality indicators was calculated by transforming the data in table 3 according to equation (2.7), and the maximum IAQI was considered to be the AQI. The AQI cloud model of Shenyang was then established using the backward cloud generator with numerical characteristics (99.59, 47.93, 27.29). A comparison of the AQI from the cloud models and corresponding standard cloud models is shown in figure 3.

The qualitative concept of AQI fell between Moderate and Lightly Polluted in the standard cloud model, i.e. the qualitative assessment result of AQI was between Moderate and Lightly Polluted (figure 3). The similarity between the AQI cloud model and its corresponding standard cloud models was calculated to determine the specific level to which it belonged via equation (2.5), with *λ*_2_ = 0.0129, *λ*_3_ = 0.0065 and others 0. The AQI cloud model belonged to Moderate in the standard cloud model according to the maximum membership degree principle. That is to say, the quantitative assessment result of AQI was Moderate.

The qualitative assessment result of AQI was between Moderate and Lightly Polluted, and the quantitative assessment result of AQI was Moderate. The qualitative and quantitative assessment results altogether suggest that Shenyang had a Moderate AQI air pollution level in November 2016.

The coverage area of the AQI cloud model was large, the fuzziness in determining the air pollution level was significant, and there were several days that were more seriously polluted than the expectation; the air pollution data were scattered and there were rapid changes in air pollution levels across the board (figure 3). If the AQI air pollution level is Moderate, there must have been several days that were more severely than Moderately Polluted. The data shown in table 3 also support this conclusion, as several days fell into the Heavily Polluted category. The air quality assessment results gathered by qualitative and quantitative analysis of the cloud model, and the fuzziness and randomness of the assessment results, merit thorough analysis by taking entropy *En* and hyper entropy *He* into consideration.

### 4.4. Air pollution changes over time

**Figure 4.**
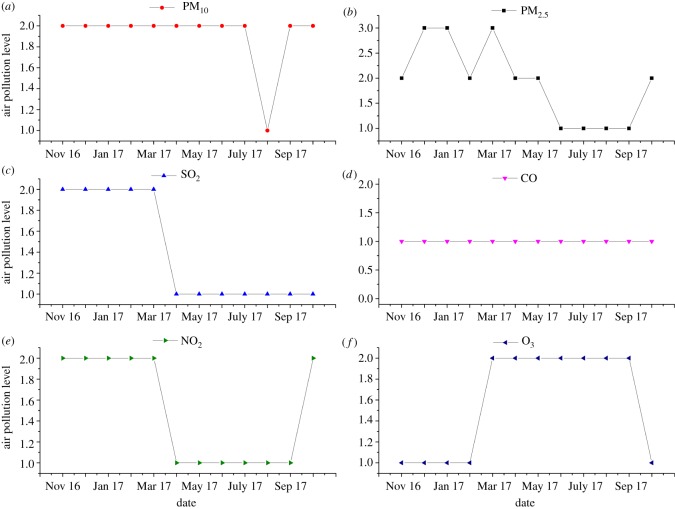
Air pollution levels over time.

Some air pollution indicators may have seasonal variations. The seasonal variation characteristics of the air pollution indicators discussed above were also assessed to explore this phenomenon. The original monitoring values of air pollution indicators (electronic supplementary material) and the cloud model and assessment results of air pollution indicators with temporal changes were determined similarly (table 4) as shown in figure 4.

The air pollution levels of PM_10_ and CO were basically unchanged with time, while air pollution levels of PM_2.5_, SO_2_ and NO_2_ were higher in winter than summer months (figure 4). Liang *et al*. also reported higher air PM_2.5_ levels in winter than summer in Shenyang [41]. Temperatures in Shenyang can drop to as low as −30°C in the winter, and heating is mainly supported by coal-fired sources which release sulfur and nitrogen oxides [42,43] as well as a large amount of dust which altogether contribute to a spike in PM_2.5_, SO_2_ and NO_2_ levels in the air. By contrast, the O_3_ air pollution level was lower in winter than summer in Shenyang. O_3_ also directly corresponds to climate change [44], as it is related to solar radiation and general atmospheric circulation, which are lower in winter than in sunny summer months. The ozone concentrations were also lower in winter than summer in Shanghai, China [45].

Seasonal variations in air pollution concentrations can provide guidance for government policy decision-makers, residents and individuals making travel arrangements. For example, as air pollution level of PM_2.5_ is higher in winter than summer, government personnel may call for adjusted heating methods such as replacing coal fire-based with electric heating systems. Individuals are also advised to wear protective masks outdoors to safeguard their respiratory systems against atmospheric pollutants.

## 5. Conclusion

The present cloud model can be successfully applied to the assessment of air quality. The cloud model correctly indicated PM_2.5_, PM_10_, SO_2_ and NO_2_ air pollutant levels to be Moderate and CO and O_3_ to be Good in Shenyang in November 2016. The cloud model was successfully validated by grey relational analysis, and the result confirmed that the cloud model can be applied to the assessment of air quality. The cloud model also showed AQI air pollution level of Moderate in Shenyang in November 2016. The fuzziness and randomness of the assessment results were effectively analysed by taking entropy *En* and hyper entropy *He* into consideration. Seasonal variation characteristics of different air pollution indicators were also obtained to find that the air pollution levels of PM_10_ and CO are consistent year-round, while the levels of PM_2.5_, SO_2_ and NO_2_ are higher in winter than summer while O_3_ is higher in summer than winter.

## Supplementary Material

Air pollution data

## Data accessibility

The Matlab code is available in Xu *et al*. [31], and the datasets supporting this paper have been uploaded as electronic supplementary material.
